# Importation and Domestic Transmission of *Shigella sonnei* Resistant to Ciprofloxacin — United States, May 2014–February 2015

**Published:** 2015-04-03

**Authors:** Anna Bowen, Jacqueline Hurd, Cora Hoover, Yvette Khachadourian, Elizabeth Traphagen, Emily Harvey, Tanya Libby, Sara Ehlers, Melissa Ongpin, J. Corbin Norton, Amelia Bicknese, Akiko Kimura

**Affiliations:** 1 Division of Foodborne, Waterborne and Environmental Diseases, National Center for Emerging, Zoonotic, and Infectious Diseases, CDC; 2 San Francisco Department of Public Health; 3 Philadelphia Department of Public Health Division of Disease Control; 4 Massachusetts Department of Public Health; 5 California Emerging Infections Program; 6 California Department of Public Health

In December 2014, PulseNet, the national molecular subtyping network for foodborne disease, detected a multistate cluster of *Shigella sonnei* infections with an uncommon pulsed-field gel electrophoresis (PFGE) pattern. CDC’s National Antimicrobial Resistance Monitoring System (NARMS) laboratory determined that isolates from this cluster were resistant to ciprofloxacin, the antimicrobial medication recommended to treat adults with shigellosis. To understand the scope of the outbreak and to try to identify its source, CDC and state and local health departments conducted epidemiologic and laboratory investigations. During May 2014–February 2015, PulseNet identified 157 cases in 32 states and Puerto Rico; approximately half were associated with international travel. Nine of the cases identified by PulseNet, and another 86 cases without PFGE data, were part of a related outbreak of ciprofloxacin-resistant shigellosis in San Francisco, California. Of 126 total isolates with antimicrobial susceptibility information, 109 (87%) were nonsusceptible to ciprofloxacin (108 were resistant, and one had intermediate susceptibility). Travelers need to be aware of the risks of acquiring multidrug-resistant pathogens, carefully wash their hands, and adhere to food and water precautions during international travel. Clinicians should request stool cultures and antimicrobial susceptibilities when they suspect shigellosis, and counsel shigellosis patients to follow meticulous hygiene regimens while ill.

*Shigella* causes an estimated 500,000 cases of diarrhea in the United States annually (1) and is transmitted easily from person to person and through contaminated food and recreational water. Outbreaks of shigellosis frequently are large and protracted. Although diarrhea caused by *S. sonnei* typically resolves without treatment, patients with mild illness often are treated with antimicrobial medications because they can reduce the duration of symptoms and shedding of shigellae in feces (2). However, resistance to the oral antimicrobial medications ampicillin and trimethoprim/sulfamethoxazole is common among shigellae in the United States, and resistance to fluorquinolones is increasing among shigellae globally (3). Because only about 2% of shigellae isolated in the United States are resistant to fluoroquinolones (4), ciprofloxacin is the first-line treatment for adults with shigellosis and is recommended as an empiric treatment for adult international travelers with diarrhea (5).

Between May 24, 2014 and February 28, 2015, PulseNet detected 157 cases of illness caused by *S. sonnei* with closely related pulsed-field gel electrophoresis (PFGE) patterns in 32 U.S. states and Puerto Rico. Most cases were reported in Massachusetts (45 cases), California (25) and Pennsylvania (18). In addition, public health officials in the San Francisco Department of Public Health (SFDPH) identified an outbreak of 95 cases of ciprofloxacin-resistant shigellosis, nine of which were tested using PFGE and have been included in the PulseNet cluster, for a total of 243 cases (Figure). The San Francisco outbreak cases are included in the antimicrobial susceptibility summary but are excluded from other analyses.

State and federal public health officials reported ciprofloxacin nonsusceptibility in 109 (87%) of 126 isolates tested (108 isolates were resistant and 1 had intermediate susceptibility). Of the 126 isolates, NARMS tested 19. All were resistant to nalidixic acid, and six (32%) were resistant to ciprofloxacin; isolates also exhibited resistance to ampicillin (5%), streptomycin (84%), sulfisoxazole (84%), tetracycline (87%), and trimethoprim/sulfamethoxazole (84%). One isolate displayed an azithromycin minimum inhibitory concentration of >256 *μ*g/ml and harbored macrolide resistance genes *mph*A and *erm*B.

Median age of the patients was 34 years (interquartile range = 20–51 years). Among the patients, 48% (74 of 153) were female. Among 41 patients with such information, median duration of illness was 7 days (interquartile range = 6–12 days). Nineteen (22%) of 88 patients with such information were hospitalized. Treatment information was not available for most patients.

Forty (53%) of 75 patients with such information had traveled internationally during their incubation period; destinations included Hispaniola (the Dominican Republic, 22 cases, and Haiti, four); India (eight); Morocco (three); and other destinations in Asia and Europe. No common airline or airport exposures were identified. Most travelers to the Dominican Republic stayed at resorts in Punta Cana; however, no common hotel, resort, restaurant, or event was reported. NARMS detected ciprofloxacin resistance in isolates obtained from travelers to the Dominican Republic (one of five isolates tested) and India (one of one isolate tested), and among nontravelers (four of seven isolates tested).

Travel information was available for 23 of 37 children; 10 (43%) had recently traveled abroad. None of the five children who were enrolled in group child care settings had traveled internationally. One pediatric case occurred as part of a child care–associated outbreak of five culture-confirmed and 11 suspected cases of shigellosis. None of the other four isolates from this cluster were tested using PFGE; however, a single isolate was tested and found to be resistant to ciprofloxacin.

Twelve patients self-identified as men who have sex with men (MSM). Eleven (79%) of 14 men without recent international travel were MSM, compared with one of six men with recent international travel (Fisher’s exact p = 0.02).

SFDPH identified 95 ciprofloxacin-resistant *S. sonnei* infections in residents of or travelers to San Francisco during November 1, 2014–January 15, 2015. Nine isolates underwent PFGE and yielded patterns that were indistinguishable from or closely related to others in the PulseNet cluster. Sixty-seven patients (53% of those with such information) were hospitalized. Seventy-four cases (47% of those with such information) occurred among persons who were homeless or living in single-room occupancy hotels. Although the investigation is ongoing, no point source or common exposures such as shelters, soup kitchens, or restaurants have been identified. No patients reported international travel.

## Discussion

International travelers are at elevated risk for colonization with multidrug-resistant *Enterobacteriaceae* (6). This investigation suggests that ciprofloxacin-resistant *S. sonnei* is being repeatedly introduced into the United States by travelers from various countries and can lead to large outbreaks domestically. The result has been a greater proportion of *Shigella* infections in the United States that are resistant to ciprofloxacin than in the past (National Antimicrobial Resistance Monitoring System; Division of Foodborne, Waterborne and Environmental Diseases; National Center for Emerging and Zoonotic Infectious Diseases, CDC, unpublished data, 2015). Travelers should be encouraged to 1) observe food, water, and hand-hygiene precautions while traveling; 2) use over-the-counter medications like bismuth subsalicylate (e.g., Pepto-Bismol) or loperamide (e.g., Immodium) if they wish to treat mild or moderate travelers’ diarrhea; 3) reserve antimicrobial medications for severe cases of travelers’ diarrhea; 4) seek health care if they are experiencing diarrhea upon return to the United States or develop diarrhea shortly thereafter; and 5) remain vigilant regarding hygiene practices while ill. Additional studies are needed to clarify the roles of antimicrobial medications, antidiarrheal medications, and other factors in acquiring multidrug-resistant enteric pathogens during international travel.

Although this *Shigella* strain is strongly associated with international travel, it is now circulating domestically. If introduced to populations of homeless persons, MSM, or children in child care settings, *Shigella* can spread rapidly and cause large, protracted outbreaks, as has occurred in the homeless population in San Francisco.

Hygiene promotion and increased access to hygiene and sanitation infrastructure among vulnerable populations such as the homeless might help prevent transmission. MSM can reduce their risk for acquiring this and other *Shigella* strains by washing their hands meticulously and by preventing fecal-oral exposures during sex (7). Health care providers should culture the stool specimens of patients with symptoms consistent with shigellosis, reculture the stool of patients who fail to improve after antimicrobial therapy, and test bacterial pathogens for antimicrobial susceptibility. Reserving antimicrobial treatment for immunocompromised patients and patients with severe shigellosis and using antimicrobial susceptibility data strategically to guide therapy might help preserve the utility of such medications. Clinical guidelines for the testing and interpretation of azithromycin susceptibility among *Shigella* spp. are needed to improve detection and management of cases of azithromycin-nonsusceptible shigellosis.

What is already known on this topic?

Approximately 500,000 cases of shigellosis occur in the United States annually. High rates of resistance to oral antimicrobial medications complicate management of patients with shigellosis; however, ciprofloxacin has remained the recommended antimicrobial treatment for adults who acquire shigellosis within the United States or while traveling internationally.

What is added by this report?

During May 2014–February 2015, a cluster of 243 cases of shigellosis in 32 states and Puerto Rico was identified; 109 (87%) of 126 isolates tested were nonsusceptible to ciprofloxacin. Ninety-five cases were part of an outbreak of ciprofloxacin-resistant shigellosis associated with the homeless population in San Francisco, California; approximately half of the remaining cases were associated with international travel. Ciprofloxacin-resistant *Shigella sonnei* is being repeatedly introduced into the United States via travelers from various countries and is circulating domestically at rates that are higher than in the past.

What are the implications for public health practice?

International travelers should be aware of the risks for acquiring multidrug-resistant pathogens, wash their hands meticulously, adhere to food and water precautions, and try to reserve antimicrobial medications for severe cases of travelers’ diarrhea. Clinicians should request stool specimen cultures and antimicrobial susceptibilities when they suspect shigellosis, carefully consider whether antibiotic treatment is necessary, and counsel shigellosis patients to follow meticulous hygiene regimens while ill. Hygiene promotion and increased access to hygiene and sanitation infrastructure might help prevent transmission among vulnerable populations.

## Figures and Tables

**FIGURE:**
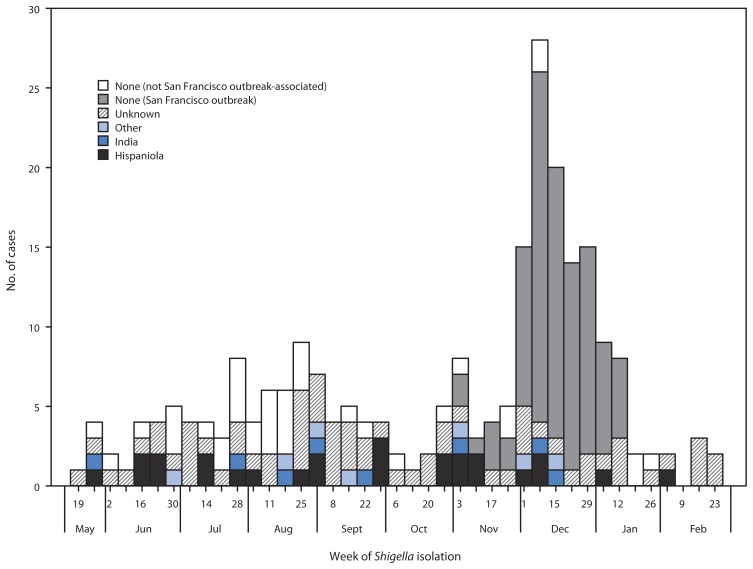
*Shigella sonnei* infections (n = 239*) suspected resistant to ciprofloxacin, by isolation date and patient international travel history — United States, May 2014–February 2015 * Isolation date was not available for four isolates.
